# A Novel Hybrid Convolutional Neural Network- and Gated Recurrent Unit-Based Paradigm for IoT Network Traffic Attack Detection in Smart Cities

**DOI:** 10.3390/s23218686

**Published:** 2023-10-24

**Authors:** Brij B. Gupta, Kwok Tai Chui, Akshat Gaurav, Varsha Arya, Priyanka Chaurasia

**Affiliations:** 1 Department of Computer Science and Information Engineering, Asia University, Taichung 413, Taiwan; 2 Center for Advanced Information Technology, Kyung Hee University, 26 Kyungheedae-ro, Dongdaemun-gu, Seoul 02447, Republic of Korea; 3 Symbiosis Centre for Information Technology (SCIT), Symbiosis International University, Pune 412115, India; 4 School of Computing, Skyline University College, Sharjah P.O. Box 1797, United Arab Emirates; 5 Department of Electrical and Computer Engineering, Lebanese American University, Beirut 1102, Lebanon; 6 Department of Electronic Engineering and Computer Science, School of Science and Technology, Hong Kong Metropolitan University (HKMU), Hong Kong; jktchui@hkmu.edu.hk; 7 Ronin Institute, Montclair, NJ 07043, USA; akshat.gaurav@ieee.org; 8 Department of Business Administration, Asia University, Taichung 413, Taiwan; 111231027@live.asia.edu.tw; 9 Center for Interdisciplinary Research, University of Petroleum and Energy Studies (UPES), Dehradun 248007, India; 10 Chandigarh University, Chandigarh 140413, India; 11 School of Computing, Ulster University, Londonderry BT48 7JL, UK; p.chaurasia@ulster.ac.uk

**Keywords:** network traffic attacks, IoT, smart cities, deep learning, CNN, GRU

## Abstract

Internet of Things (IoT) devices within smart cities, require innovative detection methods. This paper addresses this critical challenge by introducing a deep learning-based approach for the detection of network traffic attacks in IoT ecosystems. Leveraging the Kaggle dataset, our model integrates Convolutional Neural Networks (CNNs) and Gated Recurrent Units (GRUs) to capture both spatial and sequential features in network traffic data. We trained and evaluated our model over ten epochs, achieving an impressive overall accuracy rate of 99%. The classification report reveals the model’s proficiency in distinguishing various attack categories, including ‘Normal’, ‘DoS’ (Denial of Service), ‘Probe’, ‘U2R’ (User to Root), and ‘Sybil’. Additionally, the confusion matrix offers valuable insights into the model’s performance across these attack types. In terms of overall accuracy, our model achieves an impressive accuracy rate of 99% across all attack categories. The weighted- average F1-score is also 99%, showcasing the model’s robust performance in classifying network traffic attacks in IoT devices for smart cities. This advanced architecture exhibits the potential to fortify IoT device security in the complex landscape of smart cities, effectively contributing to the safeguarding of critical infrastructure

## 1. Introduction

The growth of “smart cities” is largely dependent on the IoT. Improved efficiency, sustainability, and quality of life are the results of the IoT technology that allows for the connectivity and communication of numerous objects and systems inside a city [1,2,3,4,5]. Connecting and managing household appliances and public lighting is only one example of how the IoT may be used to improve urban infrastructure and provide better services for residents. The medical applications of IoT-based systems include remote patient monitoring, effective ambulance services, and enhanced healthcare delivery [6,7,8]. The IoT facilitates the development of smart cities by easing the flow of information across disparate systems and simplifying the coordination of disparate services and devices. However, there are issues with trust and transparency that need to be resolved in order for IoT to be successfully implemented in smart cities [9]. As a whole, the IoT is essential to the growth of smart cities, as it presents a plethora of prospects for both technological advancement and environmentally responsible city planning [10,11].

The need for IoT security in smart cities is of paramount importance due to the potential risks and vulnerabilities associated with interconnected devices and systems [12,13,14]. As the adoption of the IoT in smart cities continues to grow, it is crucial to address the security challenges that arise. However, the adoption of IoT security measures in smart cities is still lagging behind [12]. Limited financial resources for investments in new physical and IoT infrastructure pose a challenge in implementing robust security measures [12]. The interconnected nature of IoT devices and systems increases the attack surface, making them susceptible to cyber threats and unauthorised access. Without adequate security measures, smart cities can be vulnerable to various risks, including data breaches, privacy violations, and the disruption of critical services. Therefore, it is essential to prioritise IoT security in smart cities to safeguard sensitive data, protect privacy, and ensure the reliable and secure operation of critical infrastructure [12,15]. Implementing strong authentication protocols, encryption mechanisms, and regular security audits can help mitigate the risks associated with the IoT in smart cities. Additionally, collaboration between stakeholders, including government bodies, technology providers, and citizens, is crucial to establish comprehensive security frameworks and guidelines for IoT deployment in smart cities [16].

In this context, we propsed a hybrid deep learning approach for the detection of cyber attack traffic in smart cities with respect to IoT. Following are our contributions: 

- Integration of Convolutional Neural Networks (CNNs) and Gated Recurrent Units (GRUs) to capture both spatial and sequential features in network traffic data, enhancing the model’s ability to identify attacks.
- Achieved an impressive overall accuracy rate of 99% after ten training epochs, demonstrating the effectiveness of the proposed approach.
- Proficiency in distinguishing various attack categories, including ‘Normal’, ‘DoS’ (Denial of Service), ‘Probe’, ‘U2R’ (User to Root), and ’Sybil’, as shown in the classification report.

The rest of the paper is organised as follows: Section 2, presents the related work and the details about our proposed work are presented in Section 3. The analysis of our propsed work is presented in Section 4. Finally, Section 5 concludes the paper.

## 2. Related Work

There are a number of tried-and-true methods for detecting attacks in the IoT, all of which are aimed at keeping the network safe from harm. Several Studies [17,18] proposes a multiclass classification approach that fits this description. The MQTT-IoT protocol is frequently used for inter-device communication; therefore, authors are investigating ways to identify attacks against it. In order to categorise network traffic and spot hostile actions, the suggested technique utilises an intrusion detection system (IDS) that makes use of machine learning methods [17,19]. The IDS is able to identify suspicious activity by comparing network packets against a baseline of known good behaviour [17,20,21]. With this method, IoT devices can be monitored and alerted in real time, which improves their security and allows for faster responses and resolutions to security events [17,22]. The findings of [17] aid in the creation of efficient attack detection techniques in IoT settings, which in turn protects IoT devices and networks from harm. Though useful, there are a number of obstacles in the application of machine learning to detect attacks in the IoT.

Based on the provided references, below are some limitations of traditional methods for attack detection in the IoT:

- Traditional approaches may not be able to keep up with the immense size and ever-changing nature of IoT networks, in which many devices produce vast volumes of data in near real-time [23].
- Traditional solutions are less effective against changing attack tactics since they depend on static rules or signatures to identify threats [24].
- Traditional approaches may produce a high number of false positives, which results in unwanted notifications and extra work for security staff [24].
- Sensor readings, network traffic, and device information are just a few examples of the many types of data that are generated by IoT networks. It is possible that conventional approaches will have difficulty analysing and comprehending such varied data [25].
- The low processing capabilities of many IoT devices make it difficult to deploy resource-intensive classical detection techniques [23].
- Delays in identifying and reacting to assaults caused by using traditional approaches might be disastrous in IoT settings, in which prompt action is required [23].
- Traditional approaches may only be able to detect anomalies that fit established attack patterns, making it difficult to identify innovative or complex attacks [26].

Table 1 present a comparative analysis of some the latest research papers. Also, Authors in [27,28,29] presents a detailed review of the application of deep learning in IoT environment. In addition to that, Refs. [30,31] presents a framework for IoT environment. From Table 1, it is clear that researchers are exploring the use of machine learning and deep learning techniques that can adapt to dynamic IoT environments, handle diverse data types, and provide more accurate and timely detection of cyber attacks [23,32].

DeepPower was proposed by Ding et al. [38] as a non-intrusive method for detecting active malware infections in IoT devices by analysing power side-channel data using deep learning. Using supervised machine learning techniques like Decision Tree, Fowdur et al. [39] explored the detection of dangerous traffic in IoT networks. In order to identify fraudulent packets in IoT settings, researchers have turned to deep learning models like LSTM and CNN. Taken together, these publications show that deep learning has promise as a method for identifying harmful behaviour in IoT systems.

## 3. Proposed Approch

### 3.1. Loss Function

In this research, we use the Cross-Entropy Loss function as a central part of our model’s optimisation. This loss function is crucial in determining the extent to which an attack categorization on network data deviates from the actual labels. Our model learns to discriminate between “Normal” and other types of attacks by minimising this loss during the course of training. Our deep learning-based solution to detecting attacks on networks carrying data from Internet of Things devices in the context of smart cities is underpinned by the Cross-Entropy Loss function. The loss function is calculated by the following equation [40]:

(1) $$\begin{array}{cc} l(x,y) & =L=\{l_{1},\cdots ,l_{N}\} \end{array}$$

(2) $$\begin{array}{cc} l_{n} & =-\sum_{c=1}^{C}w_{c}\log\frac{exp(x_{n,c})}{\sum_{i=1}^{C}exp\left(x_{n,i}\right)} \end{array}$$

where ‘x’ is the input, ‘y’ is the target, ‘w’ is the weight, ‘C’ is the number of classes, and ‘N’ spans the batch dimension.

### 3.2. Optimiser

In this research, the Adam optimizer [41,42] played a significant role in our learning process. Adaptive Moment Estimation, or Adam for short, is a widely used and very effective optimisation approach for deep learning models. Because it is a hybrid of the RMSprop and Momentum optimisers, it can update model parameters quickly and accurately using gradients. Adam is well-suited for tasks like network traffic assault detection in IoT devices inside smart cities because of its dynamically adjustable learning rates for each parameter during training. As a consequence of this flexibility, the optimizer is better equipped to deal with dynamic loss landscapes, leading to quicker convergence and higher overall model performance. Using the Adam optimizer was critical in honing our deep learning architecture and improving the model’s sensitivity to assaults in network traffic. Adam’s algorithm is presented in Algorithm 1 [41,42].
**Algorithm 1:** Adam algorithm
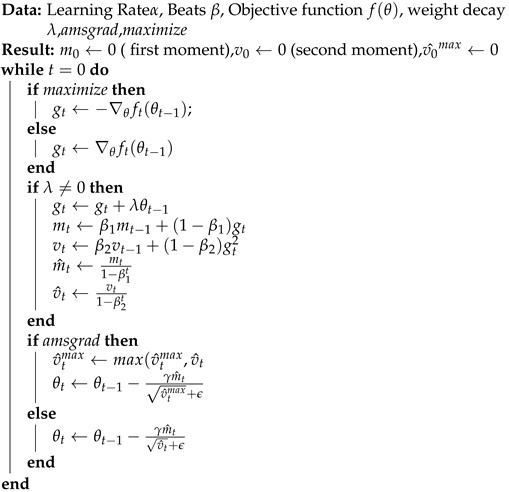


### 3.3. Model Architecture

**Figure 1:**
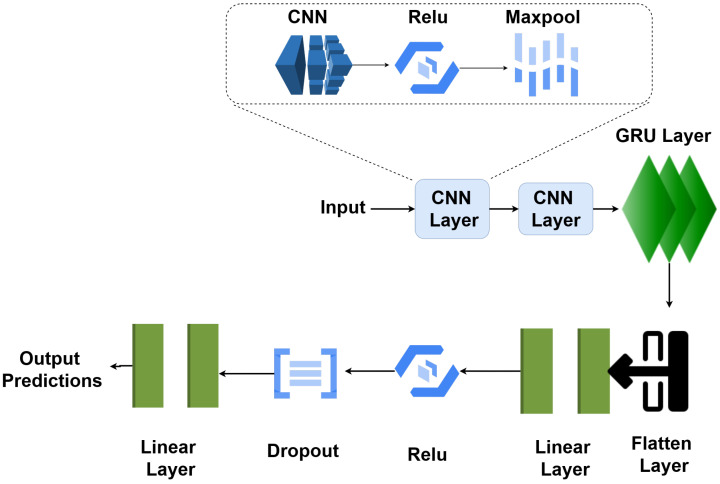
Model architecture.

The architecture of our network traffic attack detection model is presented in Figure 1. This model is a deep learning architecture that combines Convolutional Neural Networks (CNNs) and Gated Recurrent Units (GRUs) to effectively detect different types of network traffic attacks within the context of IoT devices in smart cities.

The model consists of several layers that are organised sequentially, as follows:

- The initial layer, labelled ‘DeepLearning’, represents the overall architecture.
- The first layer is a 1D convolutional layer (‘Conv1d’) with a depth of 32 and is designed to extract features from the input data. This layer has 1344 parameters.
- The ‘ReLU’ activation layer follows the convolutional layer, introducing non-linearity to the model.
- Next is a ‘MaxPool1d’ layer, which performs max-pooling to downsample the data and reduce its spatial dimensions.
- This is followed by another convolutional layer (‘Conv1d’) which has a depth of 128, further extracting hierarchical features from the data. This layer has 4224 parameters.
- Again, a ‘ReLU’ activation layer introduces non-linearity.
- Subsequently, a ‘MaxPool1d’ layer performs max-pooling.
- This is followed by the ‘GRU’ (Gated Recurrent Unit) layer, which has 128 units. GRUs are recurrent layers that can capture sequential patterns in the data.
- The ‘Flatten’ layer reshapes the output from the previous layers into a flat vector.
- Two fully connected (‘Linear’) layers follow, one with 64 and other with 5 output units. These layers have 8256 and 325 parameters, respectively.
- ‘ReLU’ activation is applied to the first fully connected layer, introducing non-linearity.
- A ‘Dropout’ layer is included for regularization, which helps prevent overfitting.
- Finally, the last ‘Linear’ layer produces the model’s output with 5 units, corresponding to the different attack categories.

This model has a total of 14,165 parameters, all of which are trainable. It combines convolutional and recurrent layers to capture both spatial and sequential features in the network traffic data, making it well-suited for the task of network traffic attack detection in IoT devices for smart cities. The model’s architecture, as depicted in Figure 1, demonstrates its depth and complexity in effectively handling the task.

## 4. Results and Disscussion

### 4.1. Data Representation

**Figure 2:**
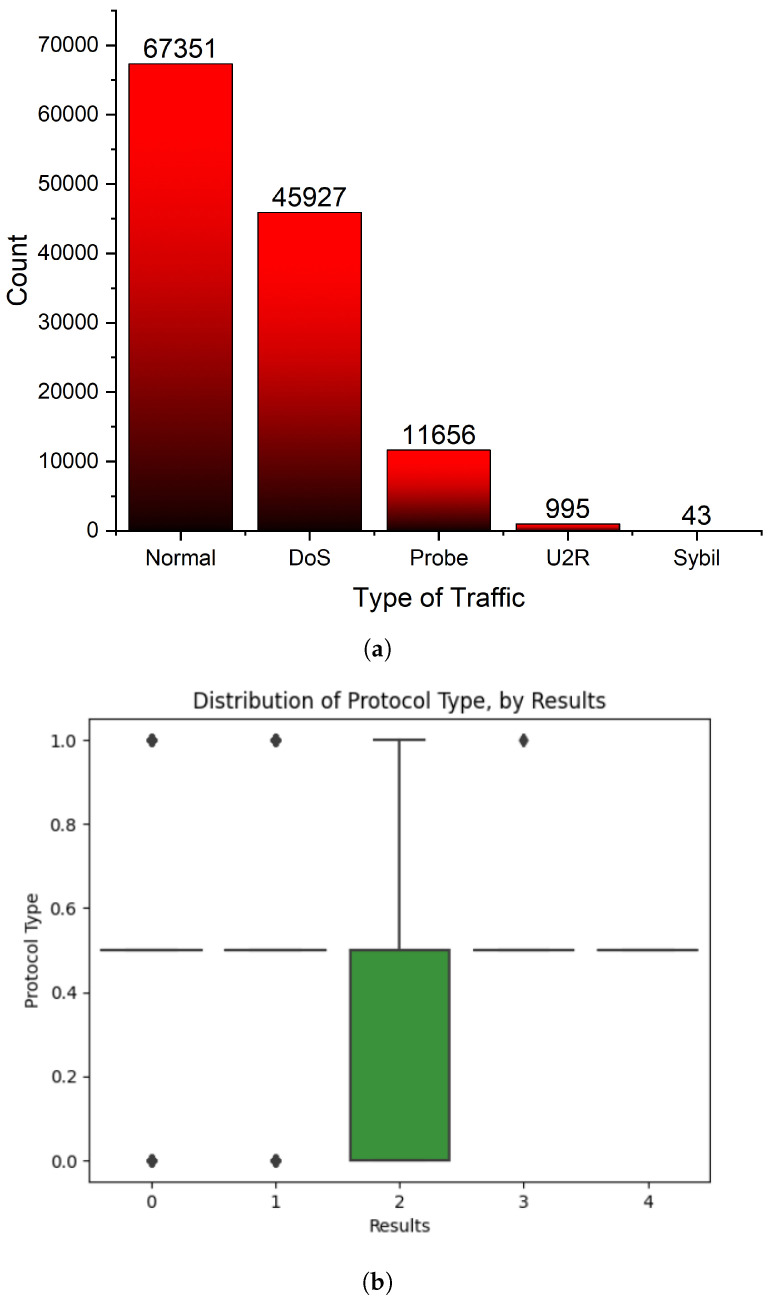

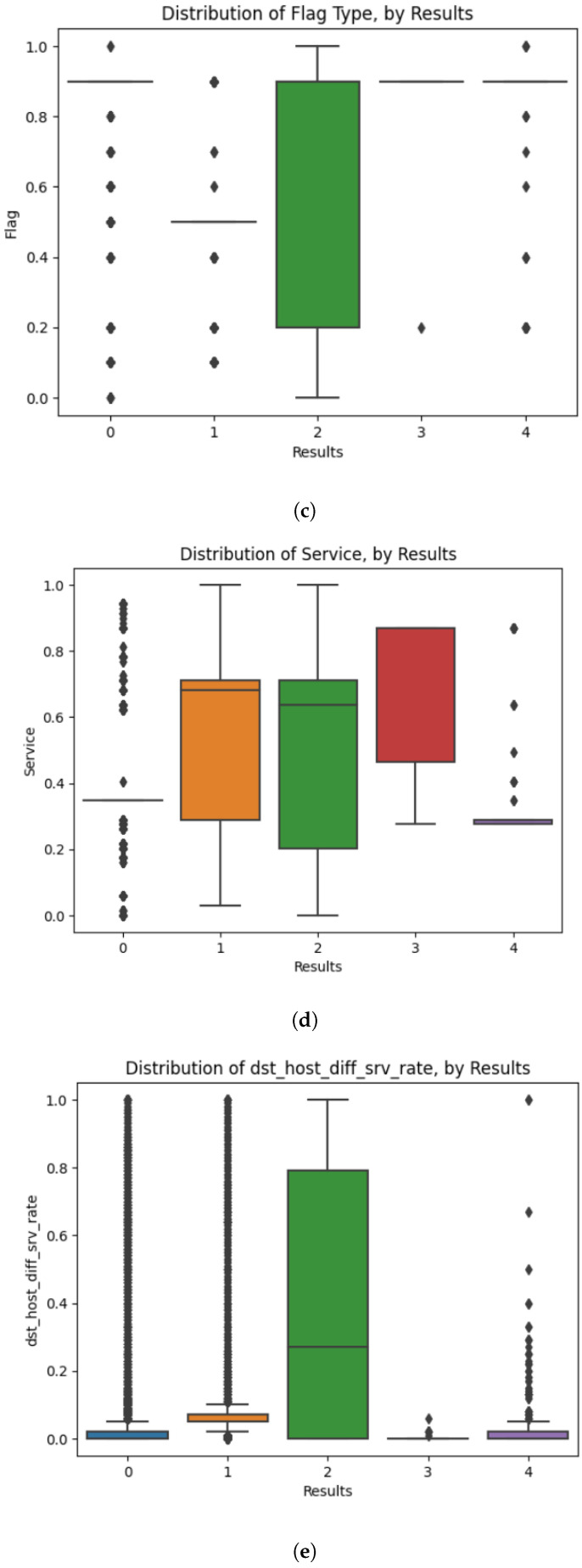

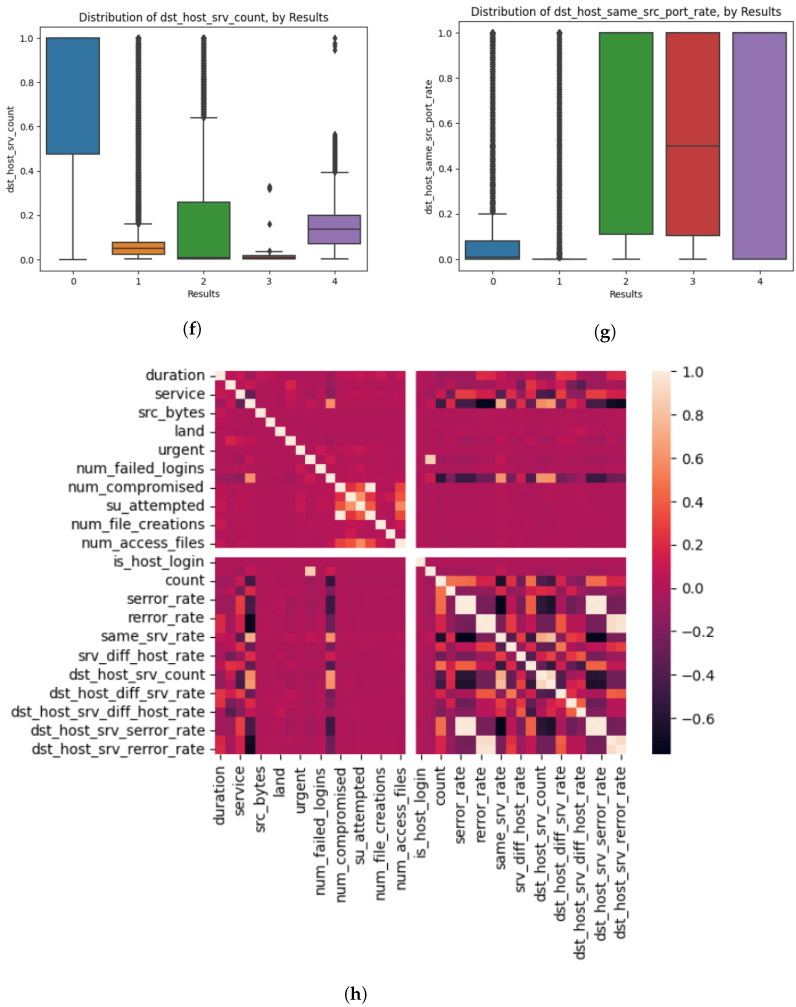
Data representaiton. (**a**) Attack map representation; (**b**) Protocol vs. attack type; (**c**) Flag vs. attack type; (**d**) Service vs. attack type; (**e**) Destination host server count vs. attack type; (**f**) Destination host server rate vs. attack type; (**g**) Destination host server port rate vs. attack type; (**h**) Correlation.

In order to construct a reliable prostate cancer detection model, we performed a thorough examination of the dataset after data preprocessing (Figure 2). This allowed us to better understand the correlations between the various variables and the target variable. Box plots, a robust visualisation tool, were used for this purpose.

### 4.2. Accuracy and Loss Curves

**Figure 3:**
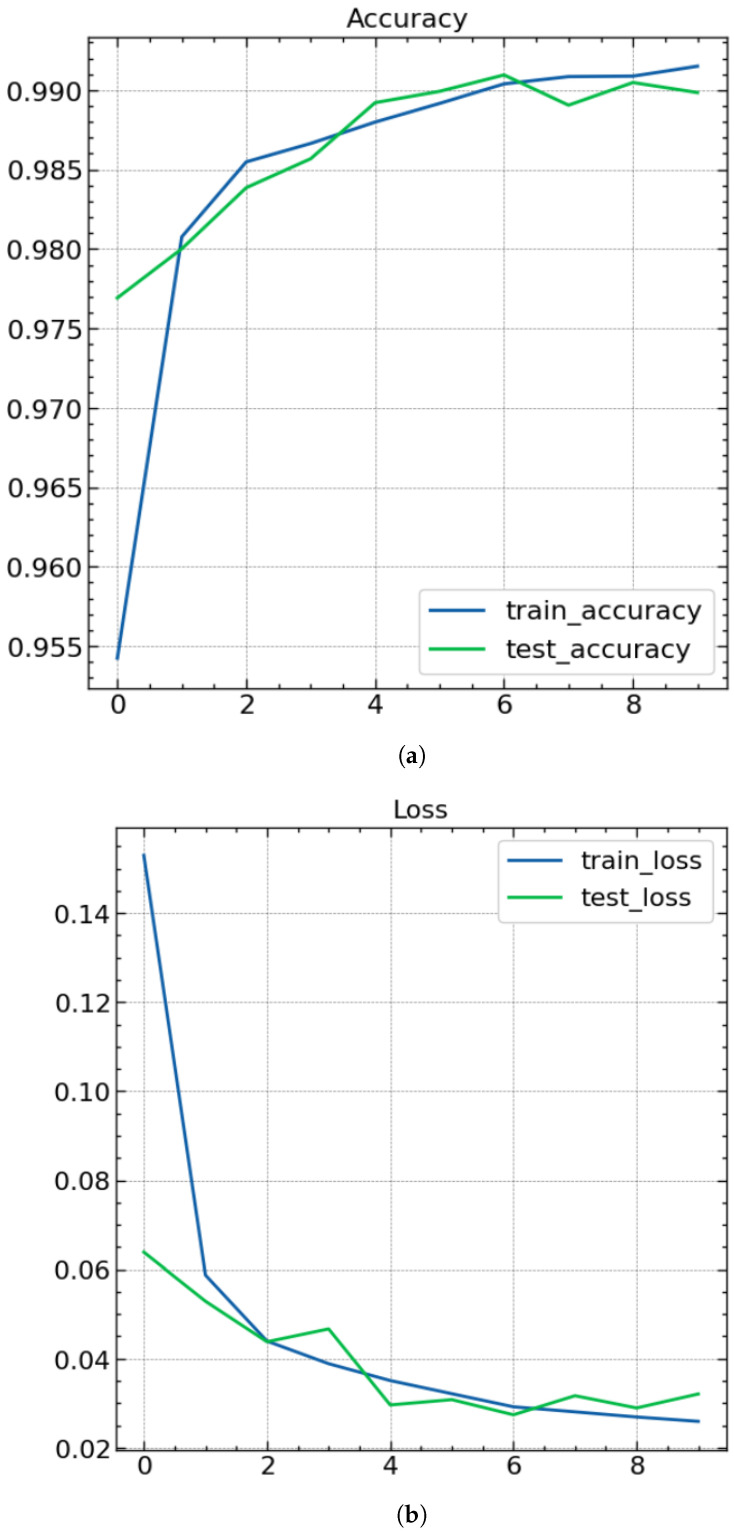
Loss and accuracy curves. (**a**) Training and test accuracy. (**b**) Training and test loss.

In this study, we conducted experiments using a Kaggle dataset to train and evaluate our CNN- and GRU-based models for the detection of network traffic attacks in IoT devices within smart cities. Our training process consisted of 10 epochs, during which we monitored the performance of our model, as represented in Figure 3. The figures below illustrate the changes in training loss, training accuracy, test loss, and test accuracy over these epochs.

As the model learnt from the data, the training loss and training accuracy both decreased from their respective values during the first epoch (0.152987 and 95.42%). To evaluate the models’ capacity to generalise, we assessed both the test loss and test accuracy simultaneously. During the initial iteration, we saw a loss of 0.063867 and an accuracy of 97.51% in our tests. Both test loss and test accuracy increased during the course of training, proving that our models are capable of identifying malicious network data. Training loss reduced steadily over all 10 epochs, demonstrating that our models improved their ability to reflect the data. Our models did not suffer from overfitting the training data, as shown by a reduction in the test loss and an improvement in the test accuracy. These findings demonstrate the promise of CNN and GRU models for protecting IoT devices in smart cities from cyberattacks.

### 4.3. Classification Report

**Figure 4:**
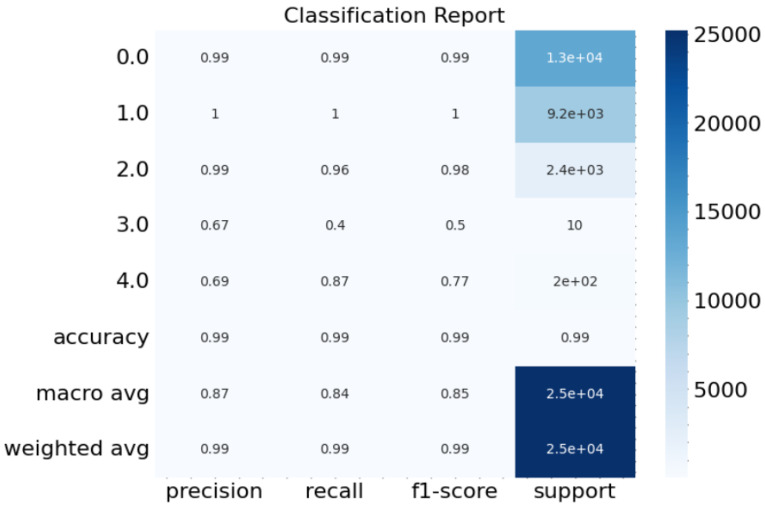
Classification Report.

In our research, we used our CNN and GRU models to identify four distinct types of network traffic attacks in the context of the IoT deployed in smart cities. The model’s effectiveness against various types of attacks is summarised in the Classification Report (Figure 4). DoS, Probe, U2R, and Sybil attacks were considered.

For each attack category, we computed three key metrics: precision, recall, and F1-score. Precision measures the accuracy of positive predictions, recall gauges the model’s ability to identify true positive cases, and the F1-score is the harmonic mean of precision and recall. These metrics provide insights into the model’s effectiveness in correctly classifying different attack types. The “support” column in the classification report represents the number of instances in each class, indicating the distribution of attack types in the dataset.

Our model’s performance varies across attack categories. It excels in distinguishing Normal and DoS attacks, with exceptionally high precision, recall, and F1-scores of 0.99 and 1.00. For Probe attacks, our model exhibits a commendable performance with an F1-score of 0.99. However, it faces challenges in classifying U2R attacks, where the F1-score drops to 0.50 due to limited support (only 10 instances). Notably, the model’s ability to detect Sybil attacks is characterised by a reasonable F1-score of 0.77, emphasising its capability to identify this specific type of attack.

In terms of overall accuracy, our model achieves an impressive accuracy rate of 99% across all attack categories. The macro-average F1-score and weighted-average F1-score are 0.85 and 0.99, respectively, showcasing the model’s robust performance in classifying network traffic attacks in IoT devices for smart cities. These results demonstrate the effectiveness of our approach in improving the security of smart city IoT networks by accurately detecting various attack types.

### 4.4. Confussion Matrix

**Figure 5:**
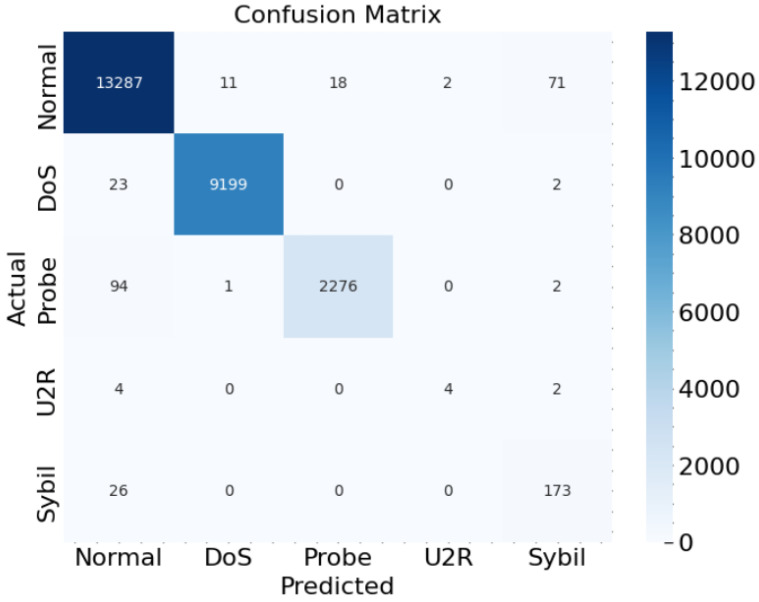
Confussion Matrix.

In our network traffic attack detection model, the confusion matrix is a valuable tool that provides a detailed breakdown of the model’s performance in classifying the different attack categories:, namely “Normal”, “DoS”, “Probe”, “U2R”, and “Sybil”. As represented in Figure 5, each row of the matrix corresponds to the true labels, while each column represents the predicted labels.

The confusion matrix illustrates the following key aspects of our model’s performance:

- For “Normal” attacks, the majority of instances (13,287) are correctly classified as “Normal”, with only a small number of instances (11) mistakenly classified as “DoS” and a few instances (18) misclassified as “Probe”. Additionally, a few “Normal” instances are incorrectly classified as “U2R” and “Sybil”, with 2 and 71 instances, respectively.
- For “DoS” attacks, the model demonstrates excellent performance, correctly classifying 9199 instances as “DoS”. There are very few false negatives (instances mistakenly classified as something other than “DoS”), with only 25 in total.
- In the case of ‘Probe’ attacks, the model correctly identifies the majority of instances (2276), with just a couple of instances misclassified as ‘Normal’ and ’U2R’.
- “U2R” attacks, being a relatively rare class with only 10 instances, have some misclassifications. Four instances are correctly classified, while six are incorrectly classified as “Normal”.
- “Sybil” attacks are correctly identified for the most part, with 173 instances correctly classified and only 26 instances mistakenly classified as ‘Normal’.

## 5. Conclusions

The security of connected devices is of critical importance in today’s ever-changing IoT and smart city scene. In this research, we provide a systematic method for dealing with the critical problem of network traffic assaults in IoT ecosystems for smart cities. Our model uses a combination of CNNs and GRUs to identify and categorise a wide variety of attacks. We have shown the efficacy of our methodology via thorough research and assessment, attaining a remarkable overall accuracy of 99%. The model’s ability to differentiate between “Normal”, “DoS”, “Probe”, “U2R”, and “Sybil” attacks was highlighted in the classification report and confusion matrix, providing significant insights into the model’s strengths and areas for development. Incorporating the findings of this study into smart city infrastructure would greatly improve the safety of IoT devices. Our model provides a reliable method for detecting malicious network traffic by combining spatial and sequential feature extraction with the capability of deep learning. Our methodology makes a substantial contribution towards this important goal as the number of smart cities grows and the necessity for robust IoT security measures becomes more pressing. In the future, networked systems will form the basis of smart cities, and it is our goal that this study can pave the way for improvements in security.

## Figures and Tables

**Table 1:** Analysis of recent papers.

Ref.	Dataset	Method	Accuracy	Precision	F1	Recall
[33]	NSL-KDD	1D-CNN	0.99	1	0.99	0.99
		2D-CNN	0.99	1	1	1
	UNSW-NB 15	1D-CNN	0.80	0.48	0.06	0.10
		2D-CNN	0.81	0.57	0.04	0.07
[34]	KDD-CUP-1999	Stochastic gradient descent classifier (SGDC)	0.9961	0.9724	0.9713	0.9718
	BotIoT-2018	SGDC	0.88	0.9403	0.9285	0.9344
	N-BaIoT-2021	SGDC	0.9691	0.9979	0.9513	0.9089
[35]	NA	Game Theory	NA	NA	NA	NA
[36]	NSL-KDD	Hybrid-CNN	0.92	0.90	0.85	0.81
[37]	OTD20	XG-Boost	0.86	1	1	1

## Data Availability

Some or all data, models, or code that support the findings of this study are available upon reasonable request from the corresponding author.
